# Polysome Profiling of a Human Glioblastoma Reveals Intratumoral Heterogeneity

**DOI:** 10.3390/ijms20092177

**Published:** 2019-05-02

**Authors:** Fernanda Cristina Sulla Lupinacci, Hellen Kuasne, Martin Roffé, Julia Avian Vassalakis, Fernanda Ferreira da Silva, Tiago Góss Santos, Victor Piana Andrade, Paulo Sanematsu, Vilma Regina Martins, Silvia Regina Rogatto, Glaucia Noeli Maroso Hajj

**Affiliations:** 1 International Research Center, A.C.Camargo Cancer Center, National Institute of Science and Technology in Oncogenomics, São Paulo 01509-010, Brazil; flupinacci@accamargo.org.br (F.C.S.L.); khellenk@yahoo.com.br (H.K.); mroffe@accamargo.org.br (M.R.); julia.vassalakis@accamargo.org.br (J.A.V.); fsilva@accamargo.org.br (F.F.d.S.); tsantos@accamargo.org.br (T.G.S.); vmartins@accamargo.org.br (V.R.M.); 2 Pathology Department, A.C.Camargo Cancer Center, National Institute of Science and Technology in Oncogenomics, São Paulo 01509-010, Brazil; victor.andrade@accamargo.org.br; 3 Neurosurgery Department, A.C.Camargo Cancer Center, National Institute of Science and Technology in Oncogenomics, São Paulo 01509-010, Brazil; psanematsu@yahoo.com.br; 4 Vejle Hospital, Institute of Regional Health Research, University of Southern, 5230 Odense, Denmark; silvia.regina.rogatto@rsyd.dk

**Keywords:** translatome, glioblastoma, tumor heterogeneity, polysome, mTORC1, translation control

## Abstract

Glioblastoma (GBM) is one of the most aggressive cancers, with median survival of less than 2 years. Despite of considerable advance in molecular classification of GBMs, no improvements in therapy have been described. The scenario is further complicated by tumor heterogeneity and the relationship among genetic, transcriptional and functional findings. Classically, gene expression has been evaluated by steady-state mRNA, however, this does not take translational control into consideration, which contributes considerably to the composition of the proteome. In this study, we evaluated the transcriptomic and translatomic signature of a GBM obtained from a single patient focusing in tumor heterogeneity. In a sampling of eight fragments, we investigated the translation rates, mTORC1 and ERK1/2 pathways and identified both total and polysome associated mRNAs. An increased translation rate was observed in fragments with high-grade histological features. High-grade histology was also associated with the expression of genes related to extracellular matrix (ECM) and angiogenesis, in both transcriptomes and translatomes. However, genes associated with epithelial to mesenchymal transition and stress response, were observed only in translatomes from high-grade fragments. Overall, our results demonstrate that isolation of translated mRNA can be used to identify biomarkers and reveal previously unrecognized determinants of heterogeneity in GBMs.

## 1. Introduction

Glioblastomas (GBMs) are tumors of the central nervous system that are among the deadliest cancers. Survival rates are of approximately 13 months with current therapeutic modalities that include surgery, radiotherapy and chemotherapy with the alkylating agent temozolomide [1]. GBMs were one of the first tumor types to be comprehensively explored by next-generation sequencing, leading to the identification of frequent genomic alterations and the development of molecular classification methods [2,3,4]. A classification based on gene expression profiles identified four molecular subtypes (Proneural, Neural, Mesenchymal, and Classical) [3]. Subsequently, upon integration of gene expression profiles and genomic alterations in GBMs and low-grade gliomas, different classes were suggested [4]. With the exception of a few genomic alterations, such as the R132H mutation in IDH1, the predictive and prognostic values of the molecular subtypes so far described are very limited [5,6].

Gene expression patterns derived from transcriptomic analysis, which correspond to steady-state mRNA levels, do not necessarily reflect the levels of proteins, the main functional product of gene expression. This fact could be one possible methodological issue contributing to the lack of a clear connection between the molecular subtypes and biological response. Gene expression is modulated at multiple levels and each layer dynamically contributes to the final proteome [7]. In particular, mRNA-translation has been suggested to modulate protein levels to a similar extent as transcription and thus is considered a fundamental post-transcriptional mechanism affecting the proteome [8,9]. In addition, mRNA translation can be selectively modulated, which results in changes in the expression of specific subsets of proteins [10]. Therefore, determining translation efficiencies of individual mRNAs in human tumors may add another layer of information that contributes to a more complete understanding of tumorigenic pathways.

Particularly in glioblastomas, important alterations of translational control pathways such as the PI3K/mTOR complex 1 (mTORC1) [11] and the phosphorylation of the α subunit of the eukaryotic translation initiation factor 2 (eIF2α) by stress-regulated kinases were observed [12,13]. The frequency of genetic alterations that may result in the activation of the PI3K/mTORC1 pathway, including but not limited to overexpression of the epidermal growth factor receptor (EGFR), mutations of PIK3CA (p110) or PIK3R1 (P85), or loss of PTEN has been estimated to affect 88% of the cases [14]. In addition, overexpression and overactivation of proteins in this pathway were also vastly documented [15]. However, to the best of our knowledge, there has been no experimental determination of the population of translated mRNAs from human GBMs.

Another layer of complexity is given by the temporal and/or spatial intratumoral molecular heterogeneity seen in GBMs [16,17,18]. Studies at single cell level demonstrated intratumoral heterogeneity both in the genome and gene expression. For example, heterogeneous amplification of EGFR and PDGFRA was observed in cell subpopulations [19,20]. Gene expression at the mRNA level also revealed populations of cells representing all of the different GBM subgroups and supported the conclusion that these tumors comprehend a heterogeneous mixture of cells [18]. These studies also present the idea that tumor heterogeneity is a possible asset to evade therapy and generate resistance, since the presence of many populations of tumor cells bearing different molecular characteristics will result in drug sensitivity heterogeneity [21,22]. In fact, studies show that low-grade gliomas and their paired recurrences display high molecular divergence [17]. 

To address these issues, we evaluated a specific transcriptomic and translatomic signature of the GBM heterogeneity at the single-patient level. We performed a subsampling of eight fragments from a human GBM tumor, evaluated translation rates, mTORC1 and ERK1/2 pathway activation and identified both total and polysome associated mRNAs. Our results demonstrate for the first time that the isolation of mRNA engaged in translation can be used to identify biomarkers of tumor progression and reveal previously unappreciated heterogeneity in diverse regulatory programs central to GBM biology, prognosis, and therapy.

## 2. Results

**Figure 1:**
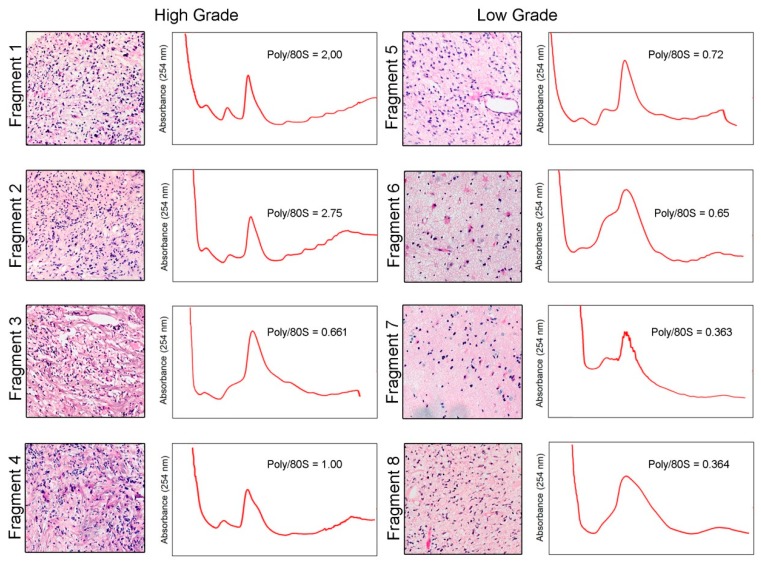
Distinct regions of a human glioblastoma (GBM) tumor present very different histological characteristics and translational rates (original magnification 10×). The tumor was dissected in eight different fragments based on macroscopical appearance of the tissue. Histological characteristics of each fragment were evaluated by HE staining and classified in high- (numbered 1 to 4) or low-grade (numbered 5 to 8). Fragments were lysed and polysome profiles were obtained by separation using ultracentrifugation in a 7–47% sucrose gradient. As a measure of translation levels, the areas under the polysome and 80S peaks were determined and the ratios of polysome/80S peak were calculated.

**Figure 2:**
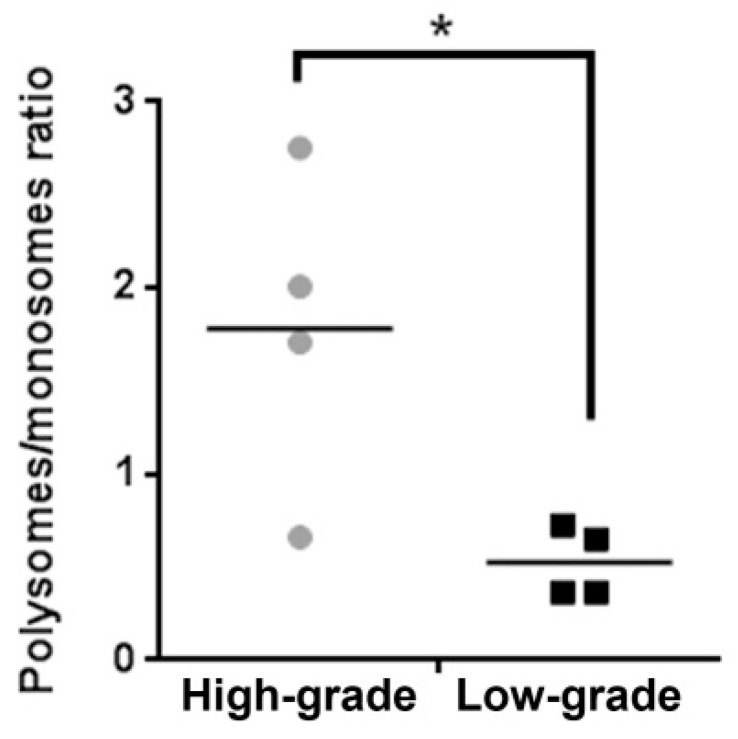
Fragments with high-grade histology have increased translation rates. Translation rates were estimated from the polysome profiles. The area under the polysome and 80S peaks was measured in Image J and the polysome/80S ratio was calculated. Fragments 1 to 4 were considered high-grade and fragments 5 to 8 were considered low-grade. * Student’s *t* test *p* = 0.02.

**Figure 3:**
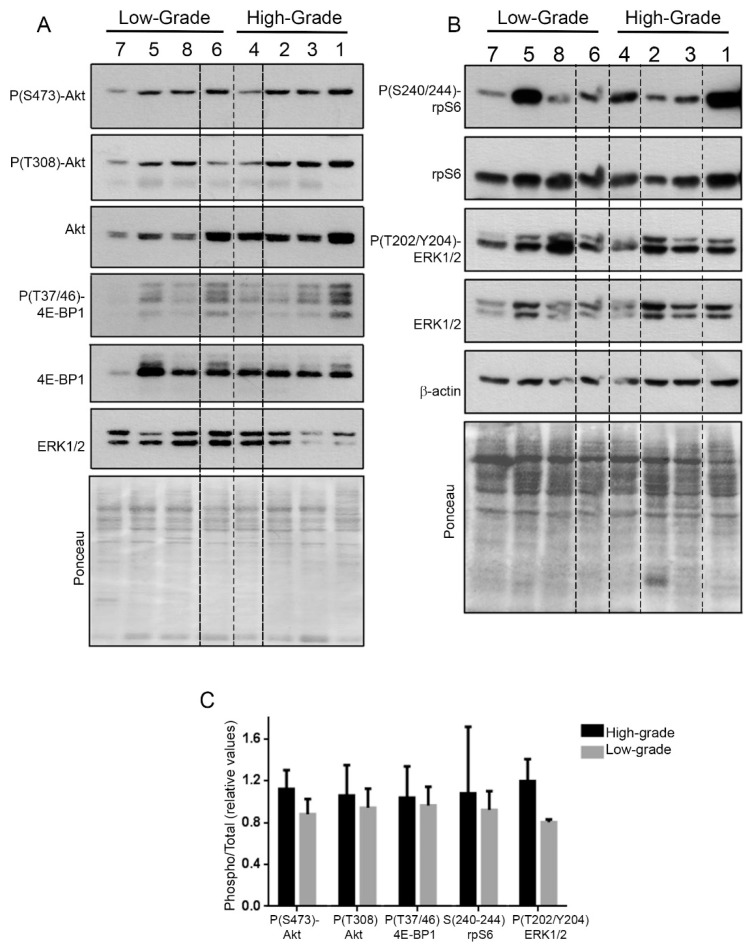
Histological grade is not associated with mTORC1 or ERK1/2 pathway activation. Tumor lysates from the eight fragments were analyzed by western blot to detect (**A**) P(S473)-Akt, P(T308)-Akt, Akt, P(Thr37/46)-4E-BP1, 4E-BP1, (**B**) P(240-244)-rpS6, rpS6, P(T202/Y204)-ERK1/2, ERK1/2 and β-actin. (**C**) Quantification was performed by ImageJ software and Phospho/total ratios were plotted.

We received eight tumor fragments from a secondary GBM case. Each fragment was subject to histopathological analysis. The histopathological analysis revealed that four of the fragments show high cellularity and pleomorphism, presence of pseudopalisades and endothelial proliferation, compatible with a high-grade histology (numbered 1 to 4), while the remaining four fragments displayed low cellularity and pleomorphism compatible with a low-grade histology (numbered 5 to 8) (Figure 1). Fragments were lysed, an aliquot of the lysate was used to extract total RNA, and the remaining was used to perform polysome profiles. Polysome profiles from high-grade fragments presented a larger area under the polysome peaks and a smaller 80S peak than low-grade fragments (Figure 1). Quantification of the polysome/80S ratio showed increase in high-grade fragments compared to low-grade ones, consistent with augmented translation rates (Figure 2). Western blot assays from the eight fragments showed that activation of the mTORC1 and ERK1/2 pathways was similar in high- and low-grade fragments. On the other hand, total levels of Akt were increased in high-grade fragments (Figure 3). Increased Akt expression in GBM when compared with low-grade gliomas has been previously documented by our group [15].

**Figure 4:**
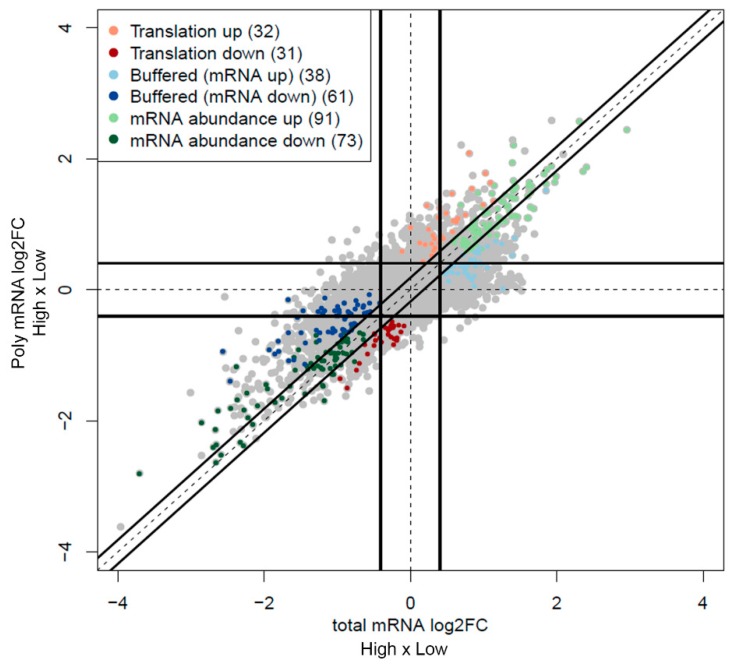
Representation of the differentially transcribed and translated genes. Microarray expression data from total and poly mRNA was analyzed by anota2seq in the comparison of high- and low-grade fragments. Gene expression values in total and poly mRNA are plotted and the regulation modes are indicated by different colors (translation—orange and red, buffering—light and dark blue, abundance— light and dark green).

We isolated RNA from the polysome profiles of all fragments. The RNA was extracted from fractions containing mRNAs associated with more than three ribosomes (>3n), and thus actively translated. We were able to obtain RNA in sufficient quality and quantity from six fragments, three high-grade and three low-grade. Total and polysome-associated mRNA (poly mRNA) expression from the six fragments were analyzed by microarray. Gene expression profiles were obtained by grouping the fragments according to their histological grade and comparing differentially transcribed and translated genes using anota2seq package (Figure 4 and Table S1) [23]. We identified a total of 326 differentially expressed genes. From a total of 161 genes up-regulated in high-grade fragments, 91 genes were by abundance (increase in both total and poly mRNA), 32 genes were by translation and 38 genes were upregulated at the transcription level but not accompanied by a change in poly mRNA (buffering). Those genes regulated by translation or buffering (70 of 161 genes or 43%) would be misidentified by using only the transcriptomic analysis. From the 165 down-regulated genes, 73 were by abundance (decrease in both total and poly mRNA), 31 genes were by translation and 61 genes were by transcription but buffered at translation. Those genes regulated by translation or buffering (92 of 165 genes or 55%) would be misidentified by using only the transcriptomic analysis.

**Figure 5:**
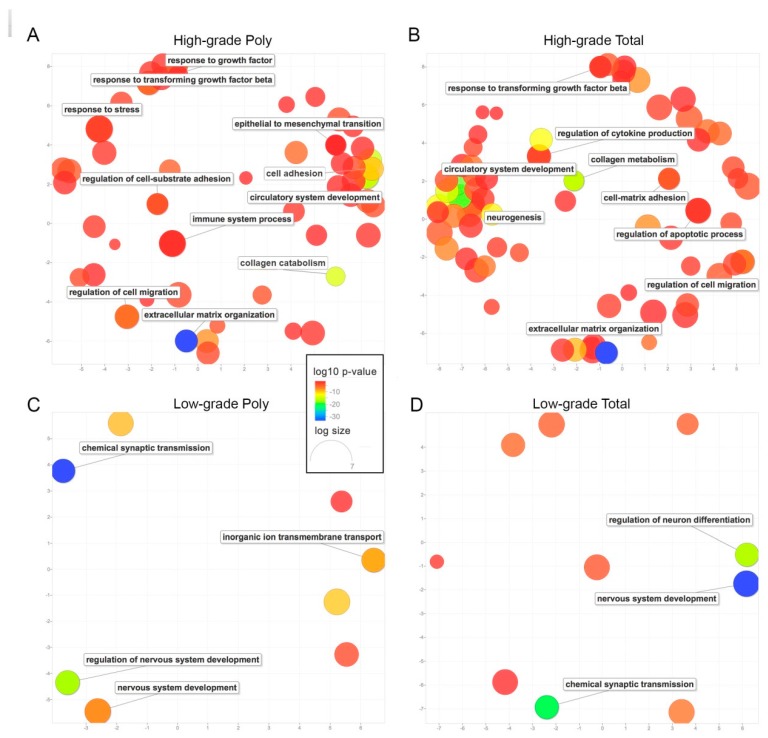
Summarization of the gene ontology (GO) processes in both total and poly RNA. GO processes obtained by STRING from the differentially expressed genes in total and poly RNA were summarized by REVIGO. The scatterplot shows the cluster representatives in a two dimensional space derived by applying multidimensional scaling to a matrix of the GO terms’ semantic similarities. *p*-values are indicated by the colors and the size of the circles indicates the frequency of the GO term in the underlying GO database. (**A**) enriched in high-grade poly mRNA, (**B**) enriched in high-grade total RNA, (**C**) enriched in low-grade poly mRNA, (**D**) enriched in low-grade total RNA.

Next, we analyzed gene ontology (GO) processes associated with differentially expressed genes in both total and poly mRNA using STRING (https://string-db.org) [24]. When looking at poly mRNA, we observed that 228 and 31 processes were found in high- and low-grade fragments, respectively (Tables S2 and S3). The total mRNA analysis revealed 311 and 28 processes in high- and low-grade fragments, respectively (Tables S4 and S5). Redundancy in GO processes was minimized and a graphical visualization was provided by REVIGO (http://revigo.irb.hr) [25] (Figure 5). In high-grade fragments, GO processes associated with ECM reorganization, collagen metabolism, circulatory system development, regulation of cell migration and response to TGF-β were observed in both total and poly mRNA (Figure 5A,B). In low-grade fragments, chemical synaptic transmission and nervous system development processes were identified in both total and poly RNA (Figure 5C,D). However, GO processes related to epithelial to mesenchymal transition and response to stress were evident only in high-grade poly mRNA (Figure 5A). Also, GO processes associated with the regulation of apoptosis and neurogenesis were observed only in total RNA, suggesting a buffering regulation (Figure 5B).

**Figure 6:**
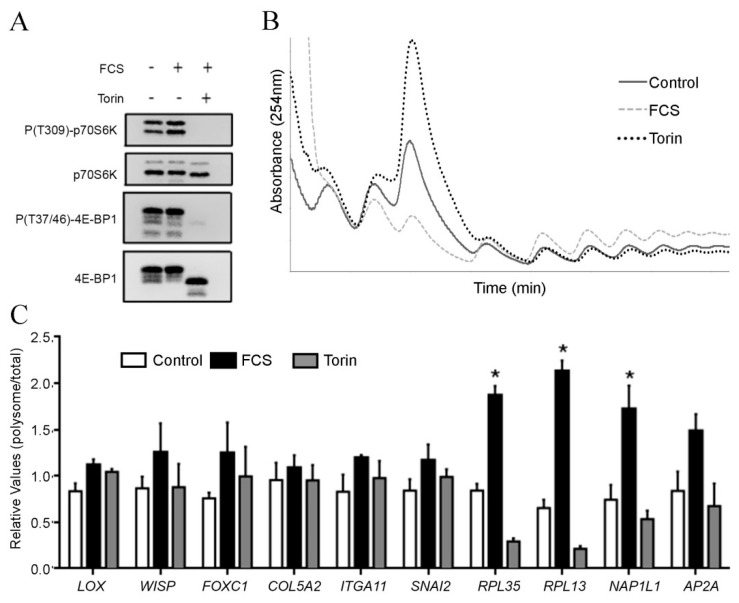
Translation of genes modulated in high-grade fragments are not dependent on mTORC1. U-87MG GBM cells were serum starved (Control), treated with serum (FCS) and serum plus the mTOR inhibitor Torin1. (**A**) Phosphorylation of mTORC1 targets (p70S6K and 4E-BP) was analyzed by Western blot as readout for mTORC1 activity. Polysome profiles were performed (**B**) and RNA was extracted and used for (**C**) qPCR for genes regulated by translation (*LOX*, *WISP*), abundance (*FOXC1*, *COL5A2*, *ITGA11*), buffering (*SNAI2*), or whose translation is regulated by mTORC1 (*NAP1L1*, *RPL13*, *RPL35* and *AP2A*). Graph displays the polysome/total ratio of expression for each mRNA. * *p* < 0.05, One Way ANOVA followed by Tukey’s *t* test.

To check if genes found in our analysis were subject to an mTORC1 dependent translational regulation, we analyzed the effects of the mTOR inhibitor Torin1 in GBM cells (Figure 6). Phosphorylation of mTORC1 targets, p70S6K and 4E-BP1, was inhibited by Torin1 treatment (Figure 6A), confirming the effectiveness of the treatment. The polysome profiles of cellular extracts revealed an increase in translation rate in FCS-treated cells that was reduced by Torin1-treatment, as expected (Figure 6B). Poly (>3n) and total RNA were extracted and subjected to qPCR. Genes previously known to be regulated by mTORC1 (*NAP1L1*, *RPL13*, *RPL35* and *AP2A*) were increased in polysomes from FCS treated cells and reduced in polysomes from Torin1 treated cells (Figure 6C). In contrast, none of the genes found in tumor fragments to be regulated by translation (*LOX*, *WISP*), abundance (*FOXC1*, *COL5A2*, *ITGA11*) or buffering (*SNAI2*) were modulated by mTORC1 in U87-MG cells (Figure 6C).

**Figure 7:**
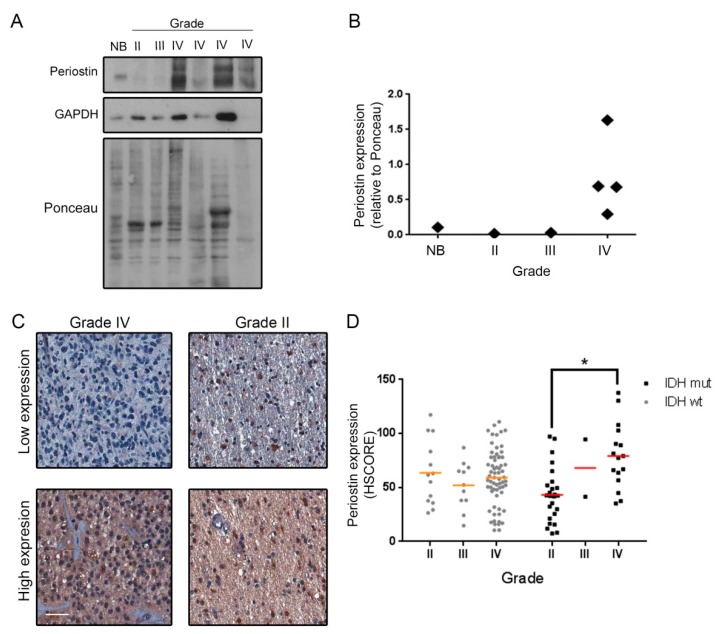
Periostin expression increases in GBMs. (**A**) Seven samples of gliomas of different grades were lysed and Periostin expression was analyzed by Western Blot. (**B**) Quantification of western blots was performed by Image J software. (**C**) Periostin expression was also evaluated by IHC in a TMA containing 138 glioma cases. (**D**) Automated quantification was performed and graph shows Periostin expression in gliomas of different grades (II, III and IV) and IDH1 status (IDH1 wild-type— IDHwt; R132H mutant IDH1— IDHmut). Scale Bar— 50 µm. * *p* = 0.004, One Way ANOVA followed by Tukey’s *t* test.

Next, we investigated the expression of Periostin (*POSTN*), whose mRNA was found modulated in our analysis of the fragments. Periostin was previously described as involved in GBM progression, resistance to antiangiogenic therapies and immune infiltration [26,27]. Periostin protein expression was evaluated by both western blot and in a tissue microarray (TMA) composed by 138 glioma tumor samples of different histological grades. The western blot analysis revealed that Periostin expression is higher in GBM samples than in the low-grade tumors (Figure 7A,B). This result was confirmed by immunohistochemistry (IHC) against Periostin in the TMA. Periostin showed increased expression in GBMs, particularly in those having the R132H mutation in IDH1, a characteristic frequently found in secondary GBMs (Figure 7C) [28]. Interestingly, our initial case of study was a secondary GBM, diagnosed first as a grade III glioma that later on progressed to a GBM. Thus, we believe that our results can provide insights to fundamental aspects of the progression from low-grade gliomas to GBMs.

Overall, our results demonstrate that intratumoral heterogeneity in GBM is marked by both transcriptional and translational events. Processes such as ECM organization and angiogenesis were a characteristic of high-grade fragments and were modulated by both transcription and translation. However, processes like epithelial to mesenchymal transition and stress response were identified only in poly mRNAs. In this specific tumor, we identified a marked activation of the ERK1/2 and mTORC1 pathways in both low- and high-grade fragments. Accordingly, none of the differentially regulated genes seemed to be sensitive to mTORC1 inhibition, suggesting that other mechanisms of translational regulation might be involved.

## 3. Discussion

This study identifies for the first time differentially translated mRNAs from different regions from a single GBM, highlighting previously unappreciated layers of information regarding gene expression control. Essential information on gene expression patterns is incomplete when looking only at the transcriptome, however, the technical difficulties associated with the isolation of poly RNA hampered its use in large scale multiomics projects [9,25]. Since poly mRNAs are engaged with the production of the respective proteins, the analysis of the translatome can provide information on gene expression patterns that more closely correlates with the expected phenotype. In our case, the modulation of genes associated with stress response or epithelial to mesenchymal transition in high-grade fragments could only be identified in poly mRNA. On the other hand, genes associated with neurogenesis were found in high-grade total RNA, suggesting that a buffering process might prevent them from being converted into proteins. We were also able to identify in the diverse regions of the tumor, differential gene expression profiles in both total and poly mRNA. In high-grade fragments, they included genes associated with ECM organization, cell adhesion, angiogenesis and TGF-β response. In low-grade fragments, several processes related to neuronal development were observed.

Two different strategies for the identification of differentially translated mRNAs are commonly implemented: polysome profiling and ribosome profiling [29], where the latter has been facilitated by the development of commercially available kits. Since translational efficiency measured by ribosome profiling has a bias towards the identification of mRNAs that show large shifts in translational efficiency, we employed polysome profiling [30]. Ribosome profiling was previously used to identify differentially translated mRNAs in a mouse GBM model in comparison with the normal brain, identifying a preferential translation of mRNAs associated with cell division and biosynthetic pathways [31,32]. Those observations were expected from a comparison between mostly non-dividing cells and highly-proliferative ones. In our case, the comparison of different tumor regions evidenced a translation program more related with tumor progression than with cell proliferation, where angiogenesis and ECM mRNAs were abundantly identified in both transcribed and translated genes. 

In the particular case of the tumor fragments of our study, there was a great variation of intratumoral status of activation of the mTORC1 and ERK1/2 pathways (Figure 3B), however this was not associated with histological characteristics or with translational rates. In fact, from the six genes chosen for validation in cellular models, none of them showed an mTORC1 dependent mode of regulation (Figure 6). Furthermore, this was concordant with the very low number of 5´TOP mRNAs [33] found in poly mRNA in the contrast between high- and low-grade. In comparison with 5’TOP lists previously published [34,35], less than 10% of poly mRNAs found to be differentially expressed in fragments were 5’TOP (Table S6). We previously reported that in secondary GBMs, the levels of mTOR, pmTOR, and P(S240–244)S6 were very similar to low-grade astrocytomas (diffuse and anaplastic) [36]. In contrast, primary GBM (IDH1 wild-type) showed increased mTORC1 pathway expression and activation when compared to wild-type low-grade gliomas. These observations suggest that in secondary GBMs mTORC1-alternative pathways of translation regulation might be relevant. The enrichment of stress-related mRNAs in polysomes from high-grade regions suggests that stress-related pathways of translation regulation, such as eIF2α phosphorylation, might be important for tumor progression. 

One very interesting target gene that was included for validation of our strategy was *POSTN*, whose product Periostin has been shown to be secreted by GBM stem cells and recruit M2 macrophages [27]. Periostin expression was increased in secondary GBMs when compared to R132H IDH1 low-grade astrocytomas (Figure 7). Accordingly, the secondary tumor we analyzed in this paper showed high POSTN levels in high-grade fragments and low POSTN levels in the low-grade ones, which is reminiscent of our results from the TMA experiment. Interestingly, Periostin expression was shown to increase under hypoxia [37], which triggers specific stress related translational control pathways [38]. Together, these data suggest that secondary GBMs may have specific tumor progression pathways related to stress response and Periostin mediated immune infiltration. 

The main limitation of our study is the use of only one tumor, which raises the possibility that the translation regulation control observed here was restricted to this case. Thus, future studies are still needed to better elucidate the role of translational control underlying GBM tumor heterogeneity. 

## 4. Materials and Methods

### 4.1. Patient

A 47-year-old woman with a previous diagnosis of anaplastic oligoastrocytoma grade III with 40% Ki-67 labelling had a surgery in another hospital. She was referred to the A.C.Camargo Cancer Center for follow up care. Before chemoradiation therapy started, recurrence was detected and the patient underwent a second surgery. After the second surgery, histopathological analysis confirmed the evolution to a secondary GBM. The patient received chemotherapy (Temozolomide) and radiotherapy after the second surgery and had a survival of 16 months after the diagnosis. We received eight histologically distinct fragments from the second surgery, which were classified as high- or low-grade, by an experienced pathologist according to the 2016 World Health Organization Classification of Tumors of the Central Nervous System [39]. The tumor was obtained with patient informed consent. Protocol was approved by the Institutional Ethics Committee in 11/12/2012 (CEP #1692/12).

### 4.2. Cells

U87-MG GBM cells (ATCC^®^ HTB-14) were maintained in DMEM plus 10% fetal calf serum (FCS). For the experiments, 2 million cells were plated and serum starved for 48 h. Cells were stimulated with FCS for 6 h with or without previous (30 min) mTOR inhibition with 250 nM Torin1 (Sigma). 

### 4.3. Polysome Profiling

The tissues were lysed in a Polytron with 400 μL of lysis buffer (20 mM Tris-HCl pH 7.5, 100 mM NaCl, 10 mM MgCl_2_, 1% Triton X-100, 1 mM DTT, 100 μg/mL cycloheximide, 100 units of RNAse inhibitor (Promega) and 1X protease inhibitor cocktail (EDTA-free)). The lysates were incubated on ice for 10 min, then centrifuged at 10.000× *g* for 10 min at 4 °C. The supernatants were collected, 60 μL were reserved for total RNA extraction and 300 µL were loaded into 7–47% sucrose gradients (20 mM Tris-HCl pH 7.5, 100 mM NaCl, 10 mM MgCl_2_, 1 mM DTT). Each gradient was formed by mixing 5.5 mL of the indicated concentration of sucrose in a Beckman Centrifuge tube (14 × 89 mm; Beckman Instruments #3311372, CA, USA) using a Labconco pump (Kansas City, MO, USA). Gradients were placed in a Beckman SW41Ti rotor and centrifuged at 39,000 rpm for 2.5 h at 4 °C. The absorbance at 254 nm was measured in a continuous flow and fractions were collected (1 mL each). Translational rates were measured by the ratio between the area under the curve of the polysome and 80S peaks, using Image J software (U. S. National Institutes of Health, Bethesda, MD, USA). 

### 4.4. RNA Extraction

Total and poly (the fractions containing the polysomes were pooled) mRNA was isolated using Trizol (Life Technologies) as described by the manufacturer. RNA integrity was verified by Agilent 2100 Bioanalyzer with an RNA Pico 6000 microfluidics kit (Agilent Technologies, CA, USA). 

### 4.5. Microarray

Gene expression in total and poly mRNA was determined using a two-color microarray-based gene expression (Whole Human Genome 4x44K microarray platform) and the Quick Amp Labeling Kit (Agilent Technologies, CA, USA) according to the manufacturer’s recommendations. RNA from the GBM cell line LN-229 (ATCC^®^ CRL-2611™) was used as reference in all reactions. Images were acquired using a DNA microarray scanner (Agilent Technologies, CA, USA) and processed by the Feature Extraction Software (v. 10.1.1.1). Microarray data are available on the Gene Expression Omnibus (GEO) database (GSE130220).

### 4.6. RT-qPCR

cDNA was synthesized using 1 μg of RNA, 0.5 μg Oligo(dT)20 Primer, 0.5 μg of random primers and GoScript™ Reverse Transcriptase. The reactions of real time PCR (qPCR) were performed using GoTaq^®^ qPCR Master Mix (Promega), according to the manufacturer’s instructions in a 7500 Real-Time PCR System (Applied Biosystems, CA, USA). Primers used are indicated in Table S7. All qPCR reactions were run in duplicate. *B2M* and *TBP1* genes were used as endogenous controls. 5’TOP mRNAs, *RPL13*, *RPL35*, *AP2A* and *NAP1L1*, were included as positive controls for mTORC1 regulation. For the data analysis, the 2^−ΔΔ*C*t^ method [40] was used and the comparative values were calculated by the ratio between expression in heavy polysomes and total RNA.

### 4.7. Data Analysis

Data from microarray were processed by the limma [41] package using the method = “loess” and bc.method = “minimum” to normalize within arrays. Probes where at least one of the samples showed a value ≤ background were discarded. The arrays were annotated according to the annotation package hgug4112a.db. Only probes with a corresponding RefSeq ID for protein (“NP”) were maintained and the annotated probes for the same mRNA were collapsed using the WGCNA [42] package keeping the probes with the higher intensities. The samples were run in three different batches and thus it was necessary to correct for batch effects using the ComBat [43] algorithm from the sva package. Since each sample was run in duplicate, we averaged the duplicates before the analysis. Normalized poly and total mRNA data were entered in the anota2seq package for R [23]. Anota2seqSelSigGenes filter was: maxSlope = 2, minSlope −1, deltaPT = log2(1.2) for translation and maxSlope = 0, minSlope = −1, deltaTP = log(1.2) for buffering; deltaP = log(1.5), deltaT = log(1.5), maxRvmP = 0.01, maxRvmPAdj = 0.70 e minEff = log(1.5). The list of differentially expressed genes in both poly and total mRNA was used as input for STRING [24] (https://string-db.org/) to identify GO processes associated with high- and low-grade. The list of GO processes was summarized and visualized using REVIGO (http://revigo.irb.hr) [25].

### 4.8. Western Blot

Cell and tissue extracts were used for western blots to detect P(S473)-Akt (#9271) 1:1000, P(T308)-Akt (#9275) 1:1000, Akt (#9272) 1:1000, P(T37/46)4E-BP1 (#9459) 1:1000, 4E-BP1 (#9452) 1:1000, P(S240-244)rpS6 (#2215) 1:1000, P(T202/Y204)-ERK1/2 (#9101) 1:1000, ERK1/2 (#9102) 1:1000 all from Cell Signaling, β-actin (A1978) 1:5000 from Sigma, Periostin (sc-67233) and rpS6 (sc-100832) 1:2000 from Santa Cruz Biotechnology, and GAPDH (MAB 374) from Millipore.

### 4.9. TMA Construction

We included 138 astrocytomas obtained from patients treated at the A.C.Camargo Cancer Center, São Paulo, Brazil, from 1980 to 2004 (Ethical Committee approval number 1485/10) [1,36] The cohort was composed by 43 diffuse astrocytomas (grade II), 13 anaplastic astrocytomas (grade III) and 82 GBMs (grade IV). No oligodendroglial or glioneuronal tumors were included. FFPE tumor tissues were spotted in a TMA containing all cases.

### 4.10. Immunohistochemistry

Immunohistochemistry was performed as described [15,43]. Briefly, TMA sections were deparaffinized and hydrated. For antigen retrieval, the slides were incubated in 10 mM citrate buffer (pH 6.0) in a pressure cooker for 30 min with preheating for 14 min. Sections were blocked for endogenous peroxidase and non-specific staining and incubated with anti-Periostin (sc-67233 Santa Cruz Biotechnology) at 1:100 dilution or anti-R132H-IDH1 (H09, Dianova) at 1:100 dilution. Secondary antibody staining was performed with the EnVision+ Dual Link kit (Dako). The primary antibody was omitted for negative controls. For Periostin quantification, an HSCORE that considers both percentage of labeling and intensity was made automatically by Aperio ScanScope XT (Wetzlar, Germany) as described [44]. Presence of staining for R132H-IDH1 was used to classify samples as wild-type, when no staining was observed, or mutant when staining of any intensity was observed. Differences in expression were tested by ANOVA followed by Tukey’s multiple comparison test. Differences were considered statistically significant at *p* < 0.05.

## Supplementary Materials

Supplementary materials can be found at https://www.mdpi.com/1422-0067/20/9/2177/s1.

## Abbreviations

4E-BP	eIF4E Binding Proteins
80S	Eukaryotic ribosomes
Akt	protein kinase B
ECM	Extracellular matrix
EGFR	Epidermal growth factor receptor
eIF2α	Eukaryotic Initation Factor 2 alpha
ERK1/2	Extracellular Signal-Regulated Kinase 1/2
FCS	Fetal Calf Serum
GBM	Glioblastoma
HE	Hematoxylin and eosin staining
mRNA	Messenger RNA
mTORC1	Mammalian Target of Rapamycin complex I
PDGFRA	Platelet-derived growth factor receptor alpha
PI3K	Phosphatidylinositol-4,5-bisphosphate 3-kinase
PTEN	Phosphatase and tensin homolog
qPCR.	Quantitative polymerase chain reaction
RNA	Ribonucleic acid
TGF-β	Transforming growth factor beta
FFPE	formalin-fixed paraffin-embedded
TMA	tissue microarray
